# Correction to ‘Recommendations for intervertebral disc notochordal cell investigation: From isolation to characterization’

**DOI:** 10.1002/jsp2.1317

**Published:** 2024-03-04

Williams RJ, Laagland LT, Bach FC, Ward L, Chan W, Tam V, Medzikovic A, Basatvat S, Paillat L, Vedrenne N, Snuggs JW, Poramba‐Liyanage DW, Hoyland JA, Chan D, Camus A, Richardson SM, Tryfonidou MA, Le Maitre CL. Recommendations for intervertebral disc notochordal cell investigation: From isolation to characterization. JOR Spine 2023;6:e1272. doi: 10.1002/jsp2.1272

The article cited above omitted an ethical review statement regarding access to human fetal samples used as examples within Figure 2 and prior development of the extractions methodology as recommended:

**Ethics Statement**

Fetal samples utilised in this study were obtained from full informed consent following medical or surgical pregnancy termination and were approved by the National Research Ethics Committee (UK) (Fetal approval number: 13/NW/0205, Paediatric approval number: 12/NW/0437).

In addition, there was a minor error in the Author Contributions; the correct text is shown below.

Rebecca J. Williams, Lisanne T. Laagland, Frances C. Bach, Lizzy Ward, Shaghayegh Basatvat, Wilson Chan, Joseph W. Snuggs, and Lily Paillat contributed to acquisition of laboratory data (Rebecca J. Williams: Porcine and Rat isolation, numeration studies, characterization, and culture; Lisanne T. Laagland: Canine and porcine isolation and characterization; Frances C. Bach: canine, and porcine isolation; Lizzy Ward: human isolation; Shaghayegh Basatvat: porcine isolation; Wilson Chan: mouse isolation; Joseph W. Snuggs: rat isolation, monolayer culture, and characterization; Lily Paillat: mouse isolation).

We apologize for this error.

